# The Physical Diagnosis of Diseases of the Lungs and Pleura

**Published:** 1890-12-20

**Authors:** 


### THE PRACTITIONER'S MIRROR AND RETROSPECT.

[*The Editor will be glad to receive offers of co-operation and contributions from members of the profession. There is no doubt that much valuable material full of interest and instruction is at present lost to science. This is largely so in the case of* (1) *the younger and less-known members of the hospital staff* (2) *those connected with cottage hospitals, and* (3) *the great body of medical practitioners not attached to any institution. The co-operation of all is cordially invited to enable the Editor to make* THE HOSPITAL *of the utmost possible use and value to the profession. Specialists willing to review books on their special subjects are invited to communicate this fact at once. All letters should be addressed to* THE EDITOR, THE LODGE, PORCHESTER SQUARE, LONDON, W.]

### THE POST-GRADUATE COURSE AT THE BROMPTON CONSUMPTION HOSPITAL.

### THE PHYSICAL DIAGNOSIS OF DISEASES OF THE LUNGS AND PLEURA.

Dr. J. Kingston Fowler, in commencing his lecture, said he proposed dealing with those points with regard to the diagnosis of some forms of chest disease which, from his long experience in the out-patient department, he considered would be of most use to his audience. He proceeded as follows: The lungs are examined in order to obtain a mental picture of the condition of the organs. In order to do this, it is essential to know the outlines of the lungs and of their respective lobes. This was necessary, to be certain of the extent of the disease in any given case. He had published elsewhere an account of what he believed to be the "march of phthisis." In this paper he had shown how the disease extends along definite lines through the pulmonary tissues. With regard to the physical examination of the chest, the first point he wished to lay stress on was *Inspection.* A careful visual examination of the chest should be made before palpation or auscultation be proceeded with. To do this satisfactorily, the patient should be undressed as much as desirable, and it was most convenient to have the patient seated on a stool, or a form without a back. The following types should then be noticed with regard to the shape of the chest, namely, the alæ, pterygoid or wing-shaped chest, that is one with short antero-posterior and lateral diameters, but long from above downwards. The front of the chest might be flat; this form was usually found in what might be called the muscular subjects of phthisis, that is, those with well-developed muscles of the shoulders and limbs. The barrel-shaped chest of emphysema. The small chest; this is especially common in women with much fat present. With regard to the emphysematous chest, the following points should be noted: the arching of the sternum, epigastric pulsation, fulness of the supra clavicular fossæ, the emphysematous girdle, which is a wavy line of injected vessels, roughly corresponding to the attachments of the diaphragm. In cases of flattening of the chest the condition of the spine posteriorly should be noted, with the patient's heels together and the head erect; possibly the occupation may have had something to do with the shape of the thorax. Another most essential point in inspection is the position of the cardiac impluse; this may be diffused, owing to the uncovering of the heart from contraction of the lungs. The movements of the chest walls should be noticed during inspiration; one shoulder may continue to move after the other. In order to note the expansion of the two sides, the fingers should be placed on the second rib; one side may continue to move after the other, or the chest may rise *en masse.* The amount of lateral expansion should also be observed behind. The following points require special attention: the arms hanging loosely down by the side; viz., the level of the inferior angles of the scapulæ (one may be lower than the other), and the distance of the vertebral borders of the scapulæ from the spine; also the difference between the two sides, with regard to expansion as seen standing behind the patient, his head bent forward. Hence the inspection of the chest should be from the front, the sides, and behind. Next, as to *Palpation,* the hands should be placed flat on the chest, the left hand on the spine posteriorly to steady the patient, and the right one in front, first on one side of the chest, and then on the other. The condition of the vocal fremitus is of great importance. It is possible in a number of cases of phthisis, even when not advanced, to determine which apex is affected from the vocal fremitus alone. Normally that on the right side is greater than that on the left. If the bronchi are not freely open the vocal fremitus is diminished. A normal standard of vocal fremitus must first be obtained by every medical man, before slight differences can be appreciated. If the vocal fremitus at each apex be marked and equal, it is probably increased at the left. If equal but feeble, possibly the right has been diminished. If at the left apex it be distinctly more marked than that at the right, there is probably disease at the left apex. If the vocal fremitus be more distinct at the right than the left, and yet the left apex is diseased, then there is often a cavity with thickened pleura over it. From these remarks the necessity of carrying on in one's mind the information derived from a previous to a further step in the physical examination must be clearly remarked. The examination must be continuous, the information derived from the various stages cumulative. If in the end all the points agree, the diagnosis should be certain.

*Percussion.*—Equally important with the change in the percussion note is the sense of resistance imparted to the fingers at the same time. With regard to the methods, it is of great importance to percuss the clavicles. Often after percussion of the first and second space the mind is left in doubt, which uncertainty may often be removed by percussion of the clavicles. The percussion finger should be allowed to remain some time on the finger used as a plexmeter, and not rebounded with a staccato-like movement. It should be remembered that the front of the chest is often more tender than the back in phthisis. The percussion generally should be light and superficial. In order to elicit differences at the bases or in the supra spinous fossæ the percussion must be firmer and deeper, and the finger in the latter situation must be pressed firmly down. The effect of inspiration on percussion should also be determined. If the underlying lung expands with inspiration, the percussion note will be altered on the patient taking a deep breath. There may be no difference in percussion of two spots during quiet breathing, yet upon deep inspiration a difference in the note may be elicited.

With regard to *Auscultation,* for the terms, it is best to use the nomenclature provisionally agreed upon by the committee appointed at the International Congress in London. First as to the breath sounds. The expressions adopted are as follows: The sound produced in the larynx is called the glottic sound, and that produced in the alveolæ known as vesicular. Over no part of the normal chest should bronchial breathing be heard, not even in the inter-scapular regions where it is termed broncho-vesicular. The bronchus does not lie, as is often supposed, in contact with the back in this region, but it is surrounded by lung tissue. A convenient table of the subject includes the following terms: vesicular, bronchial, tubular, cavernous, and amphoric. And under these heads the following conditions may be considered—pitch: quality, as compared with some normal sound; interval between inspiration and expiration; duration, of inspiration and of expiration; and the intensity (loudness). 1. *Vesicular Breathing.*—In this the pitch is low with inspiration, and lower still with expiration. Quality: inspiration vesicular, expiration blowing. Interval: none. Duration: expiration much shorter than inspiration. Intensity: inspiration variable, expiration only just heard or absent. 2. *Bronchial Breathing.*—Pitch: higher, especially of the expiratory sound. Quality: (the sound heard over the seventh cervical vertebra, that is to say, the sound produced in the glottis and conducted through the vertebra is for that chest the standard for bronchial breathing, therefore it is a tracheal sound; the inspiratory and expiratory sounds being nearly similar. Interval: distinct. Duration: expiration is equal to or is longer than inspiration. Intensity, inspiration variable, expiration greater. 3. *Tubular Breathing.*—Pitch: inspiration higher than in bronchial breathing, expiration higher still. Quality: laryngeal or whiffling, *i.e.*, that quality of breath sound heard by auscultating the larynx. Interval: distinct. Duration: inspiration equals expiration or is longer than it. Intensity: inspiration variable, expiration greater. 4. *Cavernous Breathing.*—Pitch: low; Quality: hollow or blowing, especially so the expiratory sound. Duration: expiration longer than inspiration. 5. *Amphoric Breathing.*—Pitch: low; expiration lower. Quality, hollow, metallic. Interval: distinct. Duration: expiration longer than inspiration. Intensity: inspiration variable, expiration greater.

The following terms also used were described: If the expiratory sound be prolonged, and retains its normal quality, this may be due to emphysema; if, however, it has a tracheal quality, it shows consolidation of the pulmonary tissues. *Exaggerated Breathing.*—This is of importance. If the breath sounds at one apex are harsh, and at the other feeble, it is the latter which is probably affected with disease. Feebleness of breath sounds shows some abnormal condition, simple harshness may mean increased function. This is a compensatory change, and its presence points, more or less, to a favourable prognosis. *Interrupted Breathing.*—This is often present in other conditions as well as in phthisis; it is of importance to to notice if it be present on one or both sides. *Adventitious Sounds.*—Rhonchi are common sounds, and when present it should be noticed if they are affected by coughing; sonorous rhonchi disappear on coughing, sibilant do not. Râles show the nature of the lung in the immediate neighbourhood of the lesion; they may be crackling or bubbling, and small, medium, or large. If there be consolidation or a vomica present, crackling râles are produced, if the condition be one apart from consolidation bubbling râles are heard. The term crepitation should be used for the fine crackling sounds heard in pneumonia, and in this condition only. Clicking sounds usually indicate the commencement of softening of a tubercular deposit.

After the lecture Dr. Fowler showed some interesting cases of chest diseases, one being a man with phthisis and a cardio-pulmonary murmur. The explanation of this murmur was probably the following: over the region of the heart there was some consolidated lung tissue, each systole of the heart forced out from this overlying part of the lung a certain quantity of air. This air passed through the bronchus, and was synchronous with the movements of the heart. The murmur was heard in the trachea. These murmurs may sometimes be heard by listening at a patient's open mouth. In diagnosing these murmurs aortic or mitral disease must be excluded. Many other cases of phthisis in various stages were shown, in some cases complicated with heart disease or with other pulmonary diseases, such as emphysema or pleurisy. Also a case of obstruction of the superior and inferior vena cava, probably due to mediastinitis, was demonstrated.

